# Identified or conflicted: a latent class and regression tree analysis explaining how identity constructs cluster within smokers

**DOI:** 10.1186/s40359-022-00937-y

**Published:** 2022-10-07

**Authors:** E. Meijer, W. A. Gebhardt, C. van Laar, N. H. Chavannes, B. van den Putte

**Affiliations:** 1 Public Health and Primary Care, Leiden University Medical Center, Leiden, The Netherlands; 2 National eHealth Living Lab, Leiden University Medical Center, Leiden, The Netherlands; 3 Health, Medical and Neuropsychology, Leiden University, Leiden, The Netherlands; 4 Social and Cultural Psychology, University of Leuven, Leuven, Belgium; 5 Amsterdam School of Communication Research, University of Amsterdam, Amsterdam, The Netherlands

**Keywords:** Smoking, Identity, Latent class analysis, Regression tree analysis

## Abstract

Identity, or ‘who I am’, is important for smoking behaviour. Identity constructs (parts of a person’s identity) are typically examined as separate entities, but emerging evidence suggests that the multifaceted nature of identity is relevant in the context of smoking. This cross-sectional study examined how smoking-related self- and group-identity constructs cluster within adult daily smokers (*N* = 231), whether classes of smokers can be distinguished based on clusters of identity constructs, and which factors explain class membership. Data were collected online in The Netherlands and Belgium, 2017–2018. Latent class and regression tree analyses showed that participants in Class 1 of ‘Identified smokers’ (estimated population share 54%) reported stronger smoker self- and group-identities, stronger expected identity loss when quitting smoking, and weaker quitter self-identities and non-smoker self- and group-identities (vs. Class 2 of ‘Conflicted smokers’). Class membership was explained by the interaction between mental smoking dependence (dominant explanatory variable), consideration of future consequences, age of smoking onset, self-efficacy, and future self thought clarity. Models had good fit. The identity of more dependent smokers is more strongly oriented toward smoking. Smoking is also more strongly embedded in the identity of smokers who started smoking young, are less inclined to think about the future, and have lower self-efficacy.

**Supplementary Information:**

The online version contains supplementary material available at 10.1186/s40359-022-00937-y.

## Introduction

Identity is increasingly recognized as a key factor in explaining development, maintenance and cessation of addictive behaviours, including smoking. Identity refers to perceptions of ‘who I am’, and people preferably behave in line with their identity [1–6]. The overarching concept of ‘identity’ consists of different parts, or identity constructs (e.g. ‘I am a dancer’, ‘I am someone who helps others’), that together define a person’s identity. Research into identity in the context of smoking typically focuses on self-identity and group-identity. Self-identity is defined as a part of identity that is based on a behaviour. For smokers, this entails a smoker self-identity (“Smoking fits with who I am”), non-smoker self-identity (“Non-smoking fits with who I am”), or quitting self-identity (“Quitting fits with who I am”). Group-identity refers to parts of identity that are based on memberships of social categories or groups. A strong smoker group-identity means that “being part of a group of smokers is important for who I am”, and a strong non-smoker group-identity means that the individual identifies with non-smokers. A given smoker may identify more or less strongly with each of these behaviours and groups. This study focuses on smoker, non-smoker and quitter self-identity, smoker and non-smoker group-identity, and expected identity loss when quitting smoking (see below).

Self-identity is at the basis of rules that guide an individual’s behaviour, for example a non-smoker self-identity can be accompanied by a ‘not even a puff’ rule that helps quitters in their process to refrain from smoking (PRIME theory [1]). Behaviours that are associated with identity also feel more important to individuals than behaviours that are identity irrelevant, and are therefore more likely to be performed (identity value model [3]). As such, although a sustainable health behaviour change such as quitting smoking successfully requires effortful self-control, less effort is needed when the new behaviour becomes part of identity. The individual then becomes more empowered and resilient in maintaining the new behaviour (maintain IT model [2]). In addition to such representations of behaviour in self-identity, the social identity approach elaborates on the part of the ‘self’ that is derived from group membership, and states that people are motivated to behave in line with group norms when group identification is strong and the identity is salient in a given situation [4–6]. Two related theories on overcoming addiction (the SIMOR and SIMCM models) indeed underscore the importance of smokers’ social identification with groups that support cessation [7, 8]. Many empirical studies have shown that smoker, quitter and non-smoker self-identity and group-identity are uniquely associated with intentions to quit, smoking and quitting behaviour, and reactions to antismoking measures and stigmatisation, even after other relevant variables (e.g. physical nicotine dependence) are controlled for [9–24]. In addition, it appears that the future identity as a non-smoker or quitter is more relevant for explaining smokers’ behaviour than the current identity as a smoker, suggesting that smokers need to be able to see themselves as non-smokers in order to quit smoking successfully [9–11, 23–27]. Conversely, smokers who expect or experience identity loss (i.e. smokers who feel that they lose a part of their self-identity or group-identity) after quitting are more likely to relapse [23, 28, 29].

Studies into identity and smoking or quitting typically approach identity constructs as separate entities that independently contribute to outcomes such as smoking cessation [30]. In favour of this approach, correlations between identity constructs reported in the literature show that shared variance is relatively small, such that, for example, smoker and non-smoker self-identity are not merely semantic opposites but are essentially different constructs [9, 10, 12]. Importantly, a decade ago experts advocated for investigating self-concept as ‘a dynamic and multifaceted cognitive structure’ [30], but clusters of identity constructs have not been examined to date. Nevertheless, some emerging evidence suggests that the multifaceted nature of identity is relevant in the context of smoking. For instance, although most smokers in a qualitative study did not hold strong smoker self-identities, some of them did have strong non-smoker self-identities whereas others did not [23]. Another qualitative study showed that smokers may develop incompatible group identities as both smoker and non-smoker [31]. It is likely that someone who identifies both with smoking and non-smoking (i.e. self-identity), or with both the groups of smokers and non-smokers (i.e. group-identity) behaves differently than someone who primarily identifies with only one of these behaviours or groups [23]. The term identity preference was coined to convey the relative strength of one identity construct over another, implying that not the mere strength of one identity, for example non-smoker self-identity affects behaviour, but the relative strength compared to another identity construct, such as smoker self-identity [8, 32, 33]. Importantly, physical nicotine dependence is a known and consistent predictor of the success of quit attempts [34], but recent work showed that this relationship is mediated by the relative strength of ex-smoker identity over smoker identity [35]. However, despite the growing body of literature supporting the importance of smoking-related identity in general, and emerging indications that combinations/clusters of identity constructs within one individual may be important in particular, these clusters remain unstudied.

In addition, research shows that smoking-related identity constructs are associated with other smoker characteristics, but studies typically include only a few characteristics of interest and thus a more complete picture is, as yet, lacking. With regard to demographic characteristics, two quantitative studies found that lower socio-economic position (SEP) smokers typically report stronger smoker identities than their higher SEP counterparts, and weaker non-smoker and quitter identities [9, 36], although this was not found in another study [10]. Furthermore, smoker self-identity increases, and quitter self-identity decreases over time more strongly among lower SEP smokers compared to higher SEP groups [36]. Age appears positively related to smoker self-identity and negatively related to non-smoker self-identity [11, 12], but mixed findings have been reported for gender [9–11, 20]. Smoking history and behaviour also shape identity. Smokers who started smoking at a later age, have smoked for a longer time, and those who are more dependent on nicotine or smoke more cigarettes a day have stronger smoker identities and weaker non-smoker identities [9–11, 36, 37]. Furthermore, attempting to quit and quitting smoking successfully are associated with subsequent changes in the expected direction in non-smoker, quitter, and smoker self-identity, and in smoker group-identity [12, 18, 24, 38]. The strength and development of smoker and quitter self-identity over time is also related to psychological factors such as attitudes toward smoking and quitting, quitting self-efficacy, pro-quitting social norms, and inclination to smoke in order to cope with negative emotions [12, 31, 36, 39, 40]. A qualitative study furthermore showed that smokers who perceive quitting as difficult and frightening, and who fear withdrawal symptoms, have difficulty identifying with a positive future non-smoking self [23]. In sum, previous work suggests a range of factors that relate to identity in smokers: SEP, age, gender, smoking duration, physical nicotine dependence, cigarettes per day, age of onset, quitting behaviour, attitudes and social norms, smoking as coping with negative emotions, quitting self-efficacy, and control over withdrawal symptoms. However, no studies to date have examined clusters of identity constructs within smokers and how these can be explained.

The objective of this exploratory study was two-fold. First, we examined among adult daily smokers how a range of smoking-related identity constructs cluster within individuals, and whether classes of smokers can be distinguished based on clusters of these identity constructs. We included non-smoker, quitter, and smoker self-identity, smoker and non-smoker group-identity, and expected identity loss when quitting smoking [10, 41]. Second, we examined which demographic, smoking and psychological characteristics (possibly in interaction) explain class membership. In addition to the factors mentioned above, we included clarity and frequency of thinking about the future self and consideration of future consequence, as these likely are associated with future identities as quitter and non-smoker [42–46]. Latent class analysis and regression tree analysis were used as statistical techniques.

## Method

### Design

Observational online cross-sectional study. This study reports on the pre-test measures of a longitudinal experimental study, which examined the effect of an identity-based intervention on non-smoker, quitter, and smoker self-identity and expected identity loss. These results will be reported elsewhere [47]. Data from the two follow-up measurements was not included in the current study, as these measurements were affected by the intervention. STROBE reporting guidelines were used [48].

### Participants

Participants were recruited through various means in order to reach a sufficiently large and diverse sample. They were invited after previous research participation (26%), or participated for university course credits (18%; students from two universities participated), or were recruited through social media (12%), snowball sampling (8%), a radio program (7%), approaching smokers in public places (7%), newspaper (2%), health website (3%), Google (2%), or a flyer at a cigarette point-of-sale (1%) (missing for 13%). Inclusion criteria for the larger experimental study were that participants had to be 18 years or older, smoke daily, and intend to quit some time.^1^ Participants were 231 adult daily smokers (age *M* = 37.75, *SD* = 18.53; cigarettes per day *M* = 12.07, *SD* = 8.06; 71% female; 12%, 54%, and 35% low, middle, and higher SEP, respectively). Participants were Dutch (93%) or Dutch-speaking Belgian (7%).

### Procedure

Data were collected in The Netherlands and Belgium between July 2017 and July 2018, using the Qualtrics survey program (www.qualtrics.com). Before completing the survey, participants were informed about the study aim (i.e., investigating how smokers think about smoking, quitting, themselves and the future), that participation was voluntary, and that data would be analysed and stored anonymously and treated confidentially. Two gift coupons of € 100,- and six gift coupons of € 50,- were divided among participants who also completed two follow-up questionnaires.

### Measures

Variables used in the current study are described below. There were no missing values. Bivariate correlations between identity variables and explanatory variables that were included in the final regression tree model are presented in Table 1.

**Table 1:** Descriptive statistics and Pearson correlations between variables included in the final models (*N* = 231)

Variable	M (SD)	1	2	3	4	5	6	7	8	9	10	11
1. Smoker self-identity	2.67 (0.77)	1										
2. Expected identity loss	2.27 (0.97)	0.55**	1									
3. Non-smoker self-identity	3.47 (0.70)	− 0.32**	− 0.25**	1								
4. Quitter self-identity	3.37 (0.70)	− 0.20**	− 0.23**	0.53**	1							
5. Smoker group-identity	3.40 (0.77)	0.18**	0.17**	− 0.11	− 0.06	1						
6. Non-smoker group-identity	3.45 (0.66)	− 0.17**	− 0.23**	0.20**	0.16*	0.00	1					
7. Mental dependence	2.87 (0.80)	0.36**	0.31**	− 0.24**	− 0.15*	0.07	− 0.12	1				
8. Consideration of future consequences	3.26 (0.48)	− 0.16*	− 0.25**	0.19**	0.17*	− 0.23**	0.20**	− 0.13*	1			
9. Age of onset	16.68 (3.71)	− 0.23**	− 0.10	0.10	0.17*	− 0.02	0.12	− 0.19**	− 0.04	1		
10. Self-efficacy	3.66 (0.87)	− 0.29**	− 0.28**	0.23**	0.13*	− 0.03	0.02	− 0.20**	0.04	0.07	1	
11. Future self thought clarity	2.81 (0.83)	− 0.11	− 0.19**	0.11	0.13*	− 0.09	0.06	− 0.13	0.18**	− 0.03	0.13	1
12. Physical nicotine dependence	3.06 (2.33)	0.28**	0.32**	− 0.12	− 0.11	− 0.07	− 0.01	0.40**	− 0.01	− 0.14*	− 0.36**	− 0.08

^*^p < 0.05, **p < 0.01

#### Identity

Smoker, quitter and non-smoker self-identity were measured with eight (α = 0.81), seven (α = 0.72), and seven (α = 0.80) items respectively, in order to allow for thorough measurement of these constructs (cf. [10]). Items were adapted from the Smoker Self-Concept Scale and the Abstainer Self-Concept Scale [49] and work by Tombor and colleagues [20] and Van den Putte and colleagues [12], e.g. ‘Smoking is part of “who I am”’, and ‘I can see myself as a non-smoker’. The items ‘I feel at ease with the idea that I would be a quitter/non-smoker’ in the quitter and non-smoker self-identity scales were replaced by two items adapted from the smoker self-identity scale (i.e. ‘To continue smoking fits with who I am’ and ‘To continue smoking fits with how I want to live’, [12, 24, 36]. We measured smoker (α = 0.79) and non-smoker group-identity (α = 0.68) with four items each, for example ‘In general, I am glad that I am part of the group of smokers’ (adapted from Cameron’s three factor model of group identity [50], ‘affect’ subscale). Previous work has shown that these five scales are reliable [10]. Finally, we measured expected identity loss when quitting smoking with four items, e.g. ‘If I quit smoking, I will have to give up a part of myself’ (α = 0.83, adapted from [41]). Answers ranged from [1] ‘totally disagree’ to [5] ‘totally agree’ for all items. Scales were made by calculating for each participant the mean scores across the scale items, which were then rounded to integer values for use in the latent class analysis.

#### Explanatory variables

##### Demographic characteristics

Participants reported their age and gender, and educational level as an indicator of SEP with answer categories ranging from [1] ‘no education’ to [8] ‘university’, and [9] ‘other, namely’ for which text responses were recoded into one of the eight categories (cf. [9, 10]).

##### Smoking history

Age at smoking onset, number of years they had been smoking, number of previous quit attempts, and date and duration of their most recent quit attempt (cf. [9, 10]).

##### Nicotine dependence

We used the Fagerström Test for Nicotine Dependence (FTND) to measure physical nicotine dependence [51]. Participants provided the number of cigarettes per day, which was recoded to calculate the FTND score and also used as a separate variable in the analyses. We also measured mental dependence on smoking with two items asking how much participants would miss smoking if they were to stop smoking for good ([1] ‘I wouldn’t miss it’—[4] ‘I would miss it very much’) and how important smoking is to them ([1] ‘not important’—[4] ‘very important’]) [52]. Given the correlation below 0.60, the mental dependence items were used separately in the analyses (*r* = 0.56).

##### Intention and motivation to quit

Participants were asked when (if at all) they intended to quit smoking, with answer categories [1] ‘within 1 month’, [2] ‘within 6 months’ [3] ‘within 2 years’, [4] ‘within 5 years’, [5] ‘within 10 years’, [6] ‘quit sometime ever, but not within 10 years’, [7] ‘always remain smoking, but less’; or [8] always to remain smoking, and not less’ [9, 10]. Motivation to quit was measured with one item, i.e. ‘I am motivated to quit smoking within three months’, [1] ‘totally disagree’—[7] ‘totally agree’.

##### Self-efficacy and perceived behavioural control over withdrawal symptoms

Self-efficacy was assessed with four items asking how confident participants were about being able to decrease smoking, and to quit smoking for one day, one week and one month, [1] ‘very unconfident’—[5] ‘very confident’ (α = 0.78). Perceived behavioural control over withdrawal symptoms was measured with two items, i.e., ‘If I would quit smoking…’ ‘I feel I will have control over my feelings of withdrawal from cigarettes’ and ‘I believe that I will be capable of dealing adequately with withdrawal symptoms from smoking’, [1] ‘totally agree’—[5] ‘totally disagree’ [53], *r* = 0.68.

##### Attitude toward smoking and quitting

Measured with two separate items, i.e. ‘What is your overall opinion on smoking?’ and ‘If you would quit smoking within the next 3 months, this would be…’, with [1] ‘very positive’ to [5] ‘very negative’ [36].

##### Social norms (injunctive)

Measured with one item, i.e., ‘How do you think that most of the people important to you would feel about you quitting smoking within the next 3 months?’ ([1] ‘strongly disapprove’—[5] ‘strongly approve’) [36].

##### Consideration of future consequences (CFC)

Measured with the twelve-item Consideration of Future Consequences Scale [43], translated into pre-vocational/general secondary education level Dutch [54], e.g. ‘I consider how things might be in the future, and try to influence those things with my day to day behaviour’ (α = 0.79).

##### Future self thought

Clarity of future self was measured with three items (e.g. ‘When I picture myself in the future, I see clear and vivid images’, α = 0.68) and frequency of future self thought with one item (i.e., ‘It is common for me to spend time thinking about myself as I might be in future stages of life) with answers ranging from [1] ‘not at all true for me’ to [6] ‘completely true for me’ (adapted from [42]).

##### Anxiety

General anxiety over the past two weeks was measured with three items from the Generalized Anxiety Disorder scale [55], e.g. ‘Over the past two weeks I was not able to stop or control worrying’ with [1] ‘not at all’, [2] ‘several days’, [3] ‘over half of the days’ and [4] ‘nearly every day’ (α = 0.89). Perceived control over anxiety was measured with three items from the revised Anxiety-Control Questionnaire, e.g. ‘How well I cope with difficult situations depends on whether I have outside help’, with [1] ‘totally agree’—[5] ‘totally disagree’[56]. The anxiety control items were used separately in the analyses as scale reliability was low (α = 0.53).

#### Statistical analyses

We first performed latent class analyses on the identity variables to find the optimal classes solution, which were followed by regression tree analyses to explain class membership by the explanatory variables [57]. The analyses were performed in R statistical software version 3.2.5 [58].

##### Latent class analysis

Latent class analyses were using the poLCA package [59]. The analysis aims to reduce heterogeneity in a population to a number of latent classes, i.e. existing but unobserved subgroups of participants. This fits the purpose of identifying subgroups of smokers based on how a range of smoking-related identity constructs cluster within individuals. The model aims to maximize similarity within a class and difference between the classes [60]. A series of models were fit ranging from 1 to 5 classes. We used a maximum of 1000 iterations, and repeated each analysis 100 times to decrease chances of obtaining local maxima. The models were evaluated using maximum log-likelihood (LL), Bayesian Information Criterion (BIC), Akaike Information Criterion (AIC), and relative entropy values. Lower LL, BIC and AIC values indicate better fit. The BIC takes loss of parsimony into account and has been proposed as the most accurate fit measure for basic latent class models [59]. Furthermore, relative entropy values > 0.80 indicate sufficient certainty in classification. After selection of the best fitting model, conditional probabilities were examined to interpret the classes.

##### Regression tree analysis

Regression tree analyses were performed using the Rpart and Partykit packages [61, 62]. This procedure examines in a data-driven manner whether variables interact in explaining the outcome (i.e. class membership), and searches for optimal cut-off values in explanatory variables. Regression tree analysis examines potential interactions between explanatory variables (in contrast to more traditional techniques such as logistic regression analysis that require pre-specification of interactions), and as such may lead to novel findings. At the same time, k-fold cross-validation inherent to the regression tree analysis procedure is performed to ‘prune’ the tree. The minimum number of participants per leaf was fixed at 10, and the minimum increase in fit (complexity parameter) was set at 0.0001. For the remaining parameters we used default options. The selection process of the initial, non-pruned tree was performed 1000 times. A correct classification rate (CCR) based on the final model was calculated, which was compared to the a priori CCR (i.e., all participants assigned to the largest class). We repeated the analysis without the variable that emerged as dominant in the final model in order to better understand the data.

## Results

### Latent class analysis

**Fig. 1:**
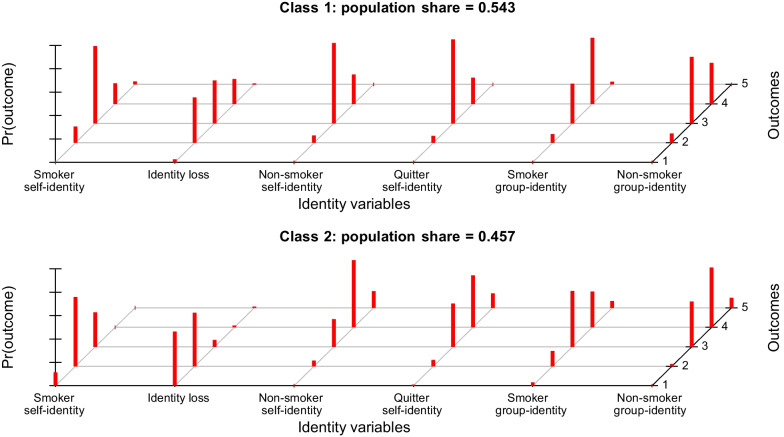
Conditional item response probabilities for the six identity variables in both classes

The model with two latent classes showed the best fit to the data according to the BIC value, which is the preferred fit measure as it takes parsimony into account (see Table 2, [59]). The relative entropy value indicated high certainty in classification (54% and 46% of the sample in Class 1 and 2, respectively). Participants in Class 1 reported stronger smoker self- and group-identities, stronger expected identity loss when quitting smoking, and weaker quitter self-identities and non-smoker self- and group-identities than participants in Class 2 (see Fig. 1 for conditional item response probabilities). The two classes differed significantly on all identity variables (see Table 3). From here on, Class 1 will be referred to as ‘Identified smokers’ and Class 2 as ‘Conflicted smokers’.

**Table 2:** Model characteristics (*N* = 231)

# Classes	LL	BIC	AIC	Parameters	Relative entropy	Class membership* (%)
1	− 1550.588	3231.794	3149.176	24	1	100
2	− 1473.268	3213.214	3044.535	49	0.9599	1: 54
						2: 46
3	− 1437.473	3277.685	3022.946	74	0.9754	1: 29
						2: 62
						3: 9
4	− 1413.718	3366.235	3025.436	99	0.9809	1: 8
						2: 13
						3: 57
						4: 22
5	− 1394.555	3463.969	3037.109	124	0.9837	1: 12
						2: 18
						3: 27
						4: 35
						5: 8

*LL *maximum log likelihood; *BIC *Bayesian information criterion; *AIC *Akaike information criterion

*Values represent estimated class population shares

**Table 3:** Scores on identity variables in the two classes: Descriptive statistics and *t*-tests (*N* = 231)

Variable	*M* (*SD*)			Mean *z*-score		*t*-test
	Overall	Class 1	Class 2	Class 1	Class 2	
Smoker self-identity	2.67 (0.77)	3.10 (0.62)	2.15 (0.60)	0.56	− 0.67	*t*(223.54) = 11.82, *p* < 0.001, *d* = 1.56
Expected identity loss	2.27 (0.97)	2.83 (0.80)	1.60 (0.70)	0.57	− 0.69	*t*(228.41) = 12.39, *p* < 0.001, *d* = 1.64
Non-smoker self-identity	3.47 (0.70)	3.18 (0.51)	3.82 (0.74)	− 0.42	0.49	*t*(179.15) = − 7.42, *p* < 0.001, *d* = 1.01
Quitter self-identity	3.37 (0.70)	3.17 (0.50)	3.62 (0.81)	− 0.29	0.35	*t*(166.57) = − 4.97, *p* < 0.001, *d* = 0.67
Smoker group-identity	3.40 (0.77)	3.56 (0.66)	3.21 (0.85)	0.20	− 0.24	*t*(194.56) = 3.39, *p* = 0.001, *d* = 0.46
Non-smoker group-identity	3.45 (0.66)	3.26 (0.60)	3.68 (0.66)	− 0.29	0.34	*t*(212.26) = − 4.97, *p* < 0.001, *d* = 0.67

Class 1 refers to ‘Identitified smokers’ (n = 126) and Class 2 refers to ‘Conflicted smokers’ (n = 105)

The three-class solution had a less favourable BIC value, and is presented in Additional file 1: Table S1. In short, in the three-class solution, Class 1 represented smokers whose smoker self-identities were oriented towards smoking, whereas Class 2 and Class 3 smokers seemed oriented towards quitting, with this pattern being more pronounced in Class 2 than 3. Group-identity seemed more important in Class 1 and 3 than in Class 2, which had most pronounced scores on self-identity. The corresponding regression tree analysis did not show predictors of class membership.

### Regression tree analysis

**Fig. 2:**
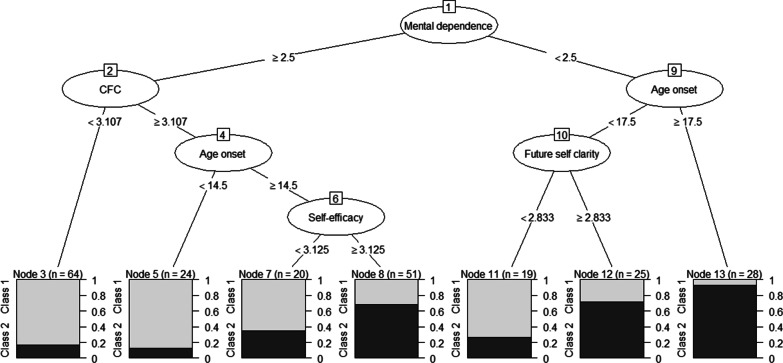
Final regression tree model. Mental dependence refers to the item ‘How much would you miss smoking if you were to stop smoking for good’

Mental dependence on smoking, consideration of future consequences (CFC), age of smoking onset, self-efficacy, and clarity of future self thought, in interaction, explained class membership, see Fig. 2 (CCR = 0.78, a priori CCR = 0.54). The other ‘explanatory variables’ (see Method, Measures) did not emerge as predictors in the regression tree analysis. Mental dependence emerged as the dominant variable. Among participants with stronger mental dependence (see Fig. 2, left side), those with weaker CFC likely belonged to Identified smokers (probability = 0.83). Among those with stronger CFC, participants who had started smoking before the age of 14.5 years likely belonged to Identified smokers (probability = 0.88). For those who were 14.5 years or older at smoking onset, self-efficacy explained class membership, such that participants with lower self-efficacy again most likely belonged to Identified smokers (probability = 0.65), but those with higher self-efficacy most likely belonged to Conflicted smokers (probability = 0.69). Participants with weaker mental dependence (see Fig. 2, right side) who had started smoking before age 17.5 were likely to belong to Identified smokers if clarity of future self thought was low (probability = 0.74), whereas those with higher clarity of future self thought were likely to belong to Conflicted smokers (probability = 0.72). Those with weak mental dependence and age of onset at age 17.5 or later were very likely to belong to Conflicted smokers (probability = 0.93).

The follow-up analysis without mental dependence showed a tree with one split on physical nicotine dependence, such that smokers with weak physical nicotine dependence were likely to belong to Conflicted smokers (FTND 0 or 1, probability = 0.66), whereas more dependent smokers were more likely to belong to Identified smokers (FTND > 1, probability = 0.64; CCR = 0.65).

## Discussion

This study provided new insight into how a comprehensive set of smoking-related identity constructs cluster within daily smokers, and how the resulting identity-based classes relate to demographic, smoking and psychological characteristics. The study confirmed emerging evidence from previous work that different identity constellations exist within smokers. Two classes emerged based on identity constructs. In short, the identity of Class 1 ‘Identified’ smokers was oriented more toward smoking, and the identity of Class 2 ‘Conflicted’ smokers was oriented more toward non-smoking, with the class of Identified smokers being only slightly larger (54%). This means that a substantial group of smokers is conflicted about their smoking, which may lead them to strongly wish to quit and become a non-smoker. Class membership was explained by (the interaction between) mental dependence on smoking, CFC, age at smoking onset, self-efficacy, and clarity of the future self. The latent class model had good fit to the data, and 78% of participants were classified correctly based on the final regression tree model (vs. 54% a priori). When the analysis was repeated without mental dependence, physical nicotine dependence explained class membership.

The extent to which smokers are dependent on smoking, both mentally and physically, seems key in explaining identity-based class membership, with smoking being more strongly embedded in identity among more dependent smokers (Identified smokers). Interestingly, mental and physical dependence shared only 16% of variance in the current study, in line with previous findings [9, 10]. Mental dependence on smoking was more important than physical nicotine dependence, which makes sense as this taps into the psychological importance of smoking [35]. Age of smoking onset also contributed to explaining class membership, both among smokers with strong mental dependence and those who were less dependent. As adolescence is typically considered as a period in which identities strongly develop [63], it is likely that teenagers who start smoking when they are younger and when their identity still needs to develop, are more susceptible to developing smoking-oriented identities (Identified smokers). Two variables concerning the future also distinguished between the two classes: consideration of future consequences (CFC; among more mentally dependent smokers) and clarity of thinking about the future self (among less mentally dependent smokers who started smoking in their teenage years). Smokers who were more oriented toward the consequences of their behaviour in the present and those who found it difficult to picture themselves in the future, respectively, were more likely to belong to the class of Identified smokers. This makes sense, as the class of Identified smokers represents an identity constellation that is more oriented in the present, with stronger (current) smoker identities, weaker (future) identities as quitter and non-smoker, and stronger expected loss of identity when quitting smoking. This finding corresponds with previous work showing that people with stronger CFC can generate more vivid images about themselves in the future and are more motivated by these future identities than lower CFC counterparts [44, 45]. Finally, self-efficacy distinguished between the identity-based classes among the specific subgroup of smokers with stronger mental dependence, relatively strong CFC, and age of smoking onset after 14.5 years, such that the identity of less self-efficacious smokers in this subgroup was more smoking-oriented (Identified smokers) whereas those with stronger self-efficacy had more non-smoking-oriented identity constellations (Conflicted smokers).

Despite well-established SEP differences in a range of smoking characteristics [64], SEP did not distinguish between the two classes in the regression tree models. Post hoc analyses in this sample showed significant SEP differences in mental dependence (the dominant variable in the regression tree), self-efficacy, and physical nicotine dependence (see Additional file 2: Table S2). It is likely that SEP is indirectly related to identity, through these variables, and therefore did not emerge as an independent explanatory variable. No significant SEP differences were found in the other explanatory variables included in the regression tree, although previous work has shown significant associations between SEP and a number of these variables [9, 64–68]. It is also possible that SEP did not explain class membership as the sample was skewed toward middle and higher SEP smokers. Several important behavioural variables did not explain class membership either, such as the number of cigarettes per day, and the number of years smoking. As for SEP, this likely results from related variables explaining class membership, and indirect associations may exist here as well. More research is warranted to fully understand how, and through which mechanisms, identity and behaviour are associated in the context of smoking.

This study has limitations. First, although a broad recruitment strategy was used, the sample was somewhat small and in some respects selective. A larger sample size would allow for thorough analysis of more complicated models with more classes, and for explaining membership of small classes [69]. The sample was not fully representative of the population of smokers as middle and higher SEP (as mentioned above), and female smokers were overrepresented. In the Netherlands in 2018, smoking was most common among those with lower SEP and among men. Specifically, about 23% of those with lower SEP were daily smokers, compared to 19% and 8% of those with middle and higher SEP, respectively. Eighteen percent of men smoked daily compared to 14% of women [70]. Relatedly, the study took place among Western European smokers. Future research may examine whether different classes emerge in other populations. Second, in order to keep survey length to a minimum, some potentially relevant variables were not included to explain class membership (e.g. current self-concept clarity [71]). Relatedly, although class membership was predicted correctly for the large majority of participants, 22% were still classified incorrectly by the regression tree model. The addition of other relevant variables might improve classification. The current study nevertheless extends previous work by being the first to examine the current selection of identity and other variables in combination. Third, the cross-sectional design prevented claims about directionality of relationships, or predictive validity. The current cross-sectional survey served as the baseline assessment of a larger longitudinal experimental study, such that participants were randomized to an future self intervention or control condition directly after completing the survey. Future observational longitudinal research may examine the direction of relationships between identity-based classes and factors that explain class membership (e.g. dependence). In addition, predictive validity of identity-based classes compared to separate identity constructs regarding smoking and quitting behaviour is as yet unknown. Fourth, certain identity constructs, or parts of identity, may be more active or salient in a given situation than others [72]. The online nature of this study prevented us from controlling the setting in which surveys were completed (e.g. at work, in a bar), but these may have affected salience of identity constructs. This in turn may have influenced strength of identity constructs as reported by participants as well as the resulting classes solution.

The current findings call for studies in different populations, and potentially different settings, to examine whether the same identity-based classes emerge. In addition, longitudinal studies are needed to assess development of identity constellations as well as class transitions within smokers over time, directionality in the relationship between identity-based classes and explanatory variables, and predictive validity of identity-based clusters regarding smoking and quitting behaviour. If explanatory variables indeed affect clusters of identity, strategies targeting for example mental dependence on smoking or consideration of future consequences may help to prevent smokers from developing identities that further complicate quitting smoking. In addition, the finding that people who started smoking at a younger age are more likely to be identified smokers provides support for increasing the legal age for selling tobacco.

Current findings also have practical implications. A substantial group of smokers is conflicted about their smoking, identifies more strongly with non-smoking and quitting than with smoking, and does not really expect to lose identity when quitting. Whereas healthcare professionals still hesitate to address smoking [73–75], this group of smokers is likely to welcome a discussion of quitting smoking and perhaps also professional smoking cessation support. Although Conflicted smokers may be ‘low hanging fruit’, smoking should also not be left undiscussed with Identified smokers. However, healthcare professionals should be careful not to threaten identity and trigger defensive or victimizing responses in this group, as was found to be a consequence of antismoking measures in smokers with weaker non-smoker self- and group-identities [9]. Optimal ways to address both groups should be studied, but in general open questions about smoking and quitting are likely to work well in starting the conversation in both groups [76]. In addition, interventions that increase non-smoker and quitter self-identity and decrease smoker self-identity, as well as help smokers to regain a complete sense of identity when experiencing identity loss during and after quitting, are potentially successful.

## Supplementary Information

**Additional file 1:** Scores on identity variables in the three classes.

**Additional file 2:** Scores on the explanatory variables in lower, middle and higher SES-groups.

## Data Availability

The data that support the findings of this study are available from the corresponding author upon reasonable request.

## Footnotes

1 Inclusion criteria were mentioned in the study information. Participants were included in the current analyses regardless of their self-reported ‘Intention to quit’.
