# Supplementary material for: Identified or conflicted: a latent class and regression tree analysis explaining how identity constructs cluster within smokers
Source: BMC Psychol. 2022 Oct 7;10:231. doi: 10.1186/s40359-022-00937-y (PMC9547436; doi:10.1186/s40359-022-00937-y)
Supplement: Supplementary file 1 — Additional file 1: Scores on identity variables in the three classes. [file 40359_2022_937_MOESM1_ESM.docx]

**Additional file 1.**

**Scores on identity variables in the three classes: Descriptive statistics (*N* = 231).**

|  | *M (SD)* | | |
| --- | --- | --- | --- |
| *Variable* | Class 1 (*N* = 64) | Class 2 (*N* = 20) | Class 3 (*N* = 147) |
| Smoker self-identity | 3.44 (0.59) | 2.05 (0.51) | 2.42 (0.62) |
| Expected identity loss | 3.23 (0.73) | 1.40 (0.99) | 1.97 (0.73) |
| Non-smoker self-identity | 2.97 (0.40) | 4.55 (0.51) | 3.54 (0.63) |
| Quitter self-identity | 3.03 (0.44) | 4.40 (1.05) | 3.38 (0.59) |
| Smoker group-identity | 3.63 (0.65) | 2.95 (1.32) | 3.36 (0.69) |
| Non-smoker group-identity | 3.11 (0.59) | 4.00 (0.79) | 3.52 (0.59) |
