# Supplementary material for: Identified or conflicted: a latent class and regression tree analysis explaining how identity constructs cluster within smokers
Source: BMC Psychol. 2022 Oct 7;10:231. doi: 10.1186/s40359-022-00937-y (PMC9547436; doi:10.1186/s40359-022-00937-y)
Supplement: Supplementary file 2 — Additional file 2: Scores on the explanatory variables in lower, middle and higher SES-groups. [file 40359_2022_937_MOESM2_ESM.docx]

**Additional file 2.**

**Scores on the explanatory variables in lower, middle and higher SES-groups: ANOVAs (*N* = 231).**

|  | *M (SD)* | | |  |
| --- | --- | --- | --- | --- |
| *Variable* | Lower  (*N* = 27) | Middle  (*N* = 124) | Higher  (*N* = 80) | ANOVA |
| Mental dependence on smoking | 3.22 (0.70)^a^ | 2.71 (0.76)^a,b^ | 2.99 (0.85)^b^ | *F*(2,228) = 6.17, *p* = 0.002, η^2^ = 0.05 |
| Consideration of future consequences | 3.17 (0.46) | 3.24 (0.45) | 3.31 (0.53) | *F*(2,228) = 1.09, *p* = 0.34, η^2^ = 0.01 |
| Age of onset | 15. 78 (2.22) | 16.91 (4.47) | 16.64 (3.71) | *F*(2,228) = 1.05, *p* = 0.35, η^2^ = 0.01 |
| Self-efficacy | 3.31 (0.81)^a^ | 3.77 (0.82)^a^ | 3.59 (0.93) | *F*(2,228) = 3.68, *p* = 0.03, η^2^ = 0.03 |
| Future self thought clarity | 2.63 (0.57) | 2.89 (0.86) | 2.75 (0.86) | *F*(2,228) = 1.37, *p* = 0.26, η^2^ = 0.01 |
| Physical nicotine dependence | 4.19 (2.20)^a^ | 2.35 (2.13)^a^ | 3.79 (2.30) | *F*(2,228) = 14.40, *p <* 0.001, η^2^ = 0.11 |
| *Note.* Subgroups of SES with the same superscript differ significantly at *p* < 0.05 (Tukey posthoc tests). | | | | |
